# Consumption of Calcium-Fortified Cereal Bars to Improve Dietary Calcium Intake of Healthy Women: Randomized Controlled Feasibility Study

**DOI:** 10.1371/journal.pone.0125207

**Published:** 2015-05-05

**Authors:** Jennifer T. Lee, Carolyn E. Moore, John D. Radcliffe

**Affiliations:** 1 Epidemiology and Genomics Research Program, National Cancer Institute, National Institutes of Health, 9609 Medical Center Drive, Rockville, MD, 20850, United States of America; 2 Department of Nutrition, Nutrition and Food Science, Texas Woman’s University, Houston, Texas, 77030, United States of America; Vanderbilt University, UNITED STATES

## Abstract

Calcium is an important structural component of the skeletal system. Although an adequate intake of calcium helps to maintain bone health and reduce the risk of osteoporosis, many women do not meet recommended daily intakes of calcium. Previous interventions studies designed to increase dietary intake of women have utilized primarily dairy sources of calcium or supplements. However, lactose intolerance, milk protein allergies, or food preferences may lead many women to exclude important dairy sources of dietary calcium. Therefore, we undertook a 9 week randomized crossover design trial to examine the potential benefit of including a non-dairy source of calcium in the diet of women. Following a 3 week run-in baseline period, 35 healthy women > 18 years were randomized by crossover design into either Group I or Group II. Group I added 2 calcium-fortified cereal bars daily (total of 400 mg calcium/day) (intervention) to their usual diet and Group II continued their usual diet (control). At the end of 3 weeks, diets were switched for another 3 weeks. Intakes of calcium and energy were estimated from 3-day diet and supplemental diaries. Wilcoxon signed-rank tests were used for within group comparisons and Mann Whitney U tests were used for between group comparisons of calcium and energy intake. Dietary calcium was significantly higher during intervention (1071 mg/d) when participants consumed 2 calcium-fortified cereal bars daily than during the baseline (720 mg/d, *P <0*.*0001*) or control diets (775 mg/d, *P = 0*.*0001*) periods. Furthermore, the addition of 2 calcium-fortified cereal bars daily for the 3 week intervention did not significantly increase total energy intake or result in weight gain. In conclusion, consumption of calcium-fortified cereal bars significantly increased calcium intake of women. Further research examining the potential ability of fortified cereal bars to help maintain and improve bone health of women is warranted.

**Trial Registration:**

ClinicalTrials.gov NCT01508689

## Introduction

Calcium is an important component of the skeletal system and an adequate intake is required to help maintain bone health and reduce the risk of osteoporosis. However, most adult women do not consume an adequate amount of this important nutrient [1]. The current recommendation for calcium is based on the amount required to maintain an adequate rate of calcium retention in bones of healthy individuals by gender and age [2]. While the Recommended Dietary Allowance (RDA) established by the Food and Nutrition Board of the Institute of Medicine is 1000 milligrams (mg) of calcium per day for women between the ages 19 to 50, and 1200 mg per day for women above age 50, most women fail to reach these intake levels [2]. Data from the National Health and Nutrition Examination Survey (NHANES) 2003–2006 indicated that 70% of women between the ages of 18 and 50 not using supplements had a total dietary calcium intake below recommended levels [1].

Maintaining an adequate calcium intake to reduce the risk of osteoporosis is especially important for women. Osteoporosis is a skeletal disorder pertaining to the deterioration of bone mass and tissues which can lead to increased bone fragility and risk of fracture. The National Osteoporosis Foundation estimated in 2014 that 9.9 million individuals in the United States had osteoporosis and 4.3 million individuals had low bone density [3]. Nevertheless, the progression of osteoporosis can be modified through changeable factors such as diet and exercise. Reducing the risk of osteoporosis includes getting enough calcium and vitamin D and engaging in regular exercise [3]. Dairy foods are the major source of calcium among adults 19+ years [4] with milk (22.5%) and cheeses (23.9%) providing the highest calcium contribution. Dairy intake, however, is significantly below the 2010 Dietary Guidelines for American [5] for many and as a result, use of non-dairy products to potentially increase calcium intake have been a focus of research [6–8].

To our knowledge, no other studies have examined calcium-fortified whole grain cereal bars as a non-dairy source of calcium. Therefore, the aim of this study was to determine the contribution of calcium-fortified whole grain cereal bars on dietary calcium intake in women. First, we hypothesized that daily consumption of calcium-fortified whole grain cereal bars would increase calcium intake of healthy women. Secondly, we hypothesized the addition of the calcium-fortified cereal bars to the usual diet of women would not significantly increase total daily calorie intake and result in weight gain. Findings demonstrated that consumption of calcium-fortified cereal bars was a feasible way to increase calcium intake of women.

## Methods

The protocol for this trial and supporting CONSORT checklist are available as supporting information; see S1 Checklist and S1 Protocol.

### Participants

The study was conducted in the Department of Nutrition and Foods Sciences, Texas Woman’s University (TWU), Houston, Texas. Forty participants were recruited through flyers posted on bulletin boards at Texas Woman’s University and several shuttle stops in the Texas Medical Center from February 2012 to April 2012 by one of the authors (JT Lee). A few participants (n = 3) contacted the study coordinator to self-recruit from the study protocol posting “Effect of calcium-fortified cereal bars on dietary calcium intake in women” registered at ClinicalTrials.gov (NCT01508689). Inclusion criteria were English speaking healthy women 18 years and older. Exclusion criteria were consumption of high dosages of calcium supplements, use of calcium-containing medications, or regular consumption of calcium fortified cereal bars. Women occasionally taking a multi-vitamin/mineral supplement containing calcium were not excluded. Women were also excluded if they were pregnant or planning to become pregnant; had liver disease, kidney disease, gastrointestinal disease (celiac disease, ulcerative colitis, or Crohn’s disease); or had a history of bariatric surgery, a major cardiovascular event (stroke or myocardial infarction), or cancer. In addition, women following a weight-controlled diet, a disease specific diet, or a vegan diet were excluded. Finally, women diagnosed with an eating disorder or with an allergy to ingredients found in the cereal bar (Kellogg’s Nutri-Grain) were excluded.

Demographic information obtained from participants included age and ethnicity. Height was measured and body weight changes were monitored during the 9 week study. The Institutional Review Board of Texas Woman’s University approved the study and written informed consent was obtained from all study participants.

### Study Design

**Fig 1:**
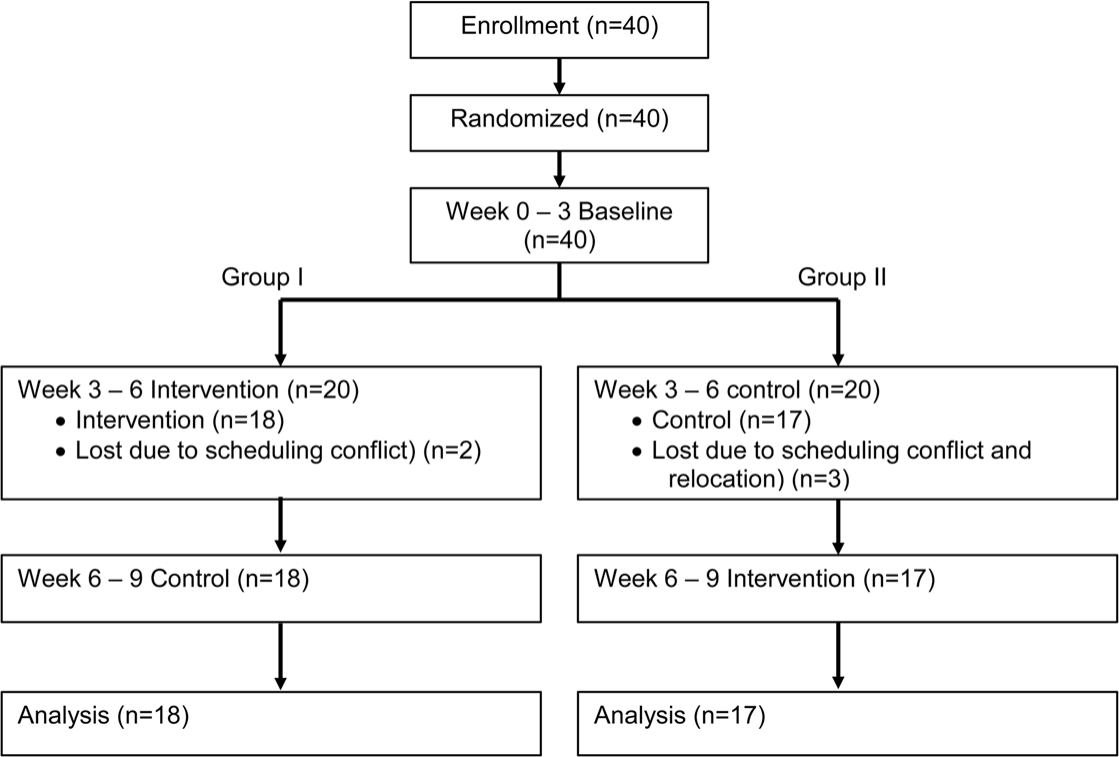
Randomized crossover study flow.

A 9 week open label randomized controlled crossover design was used. During the first 3 weeks of the study, all participants consumed their usual diet to estimate baseline energy and calcium intake. Participants were then randomly assigned into either Group I or Group II by JT Lee pulling a number out of a hat (Fig 1). During the second 3 week period, women in Group I consumed 2 Kellogg’s Nutri-Grain cereal bars (intervention) daily that provided a total of 240 calories and 400 mg of calcium [9] (Table 1). Study cereal bars were purchased at a local Houston grocery store. Women in Group II continued consuming their usual diet (control) during the second 3 week period (Fig 1). During the final 3 week period, the two study groups were switched (Fig 1). The crossover design allowed each participant to serve as her own control and helped to counterbalance any order effect. A $20 gift certificate was given to participants who completed the study. Study expenses were supported internally by the TWU Department of Nutrition and Food Sciences.

**Table 1:** Cereal bar composition.^1^

Nutrients	Amount per bar
**Calories (kcals)**	120
**Carbohydrates (g)**	24
** Dietary Fiber (g)**	3
** Sugar (g)**	11
**Protein (g)**	2
**Fat (g)**	3
**Calcium (mg) as calcium carbonate**	200

^1^ Reference [9].

### Dietary Intake

Dietary intake was recorded as 3 day diet and supplement diaries (3DS) consisting of 2 weekdays and 1 weekend day. Each participant completed a 3DS during study weeks 2, 5, and 8. Each participant was provided instructions on how to record daily intake and measuring cups were offered. Specific brand names, amount, content, and the time when each food item or supplement was consumed were recorded. Participants were also encouraged to contact the investigators to answer any questions. JL reviewed each food record with participants to confirm completeness of information and to verify amounts consumed. The average energy and calcium intake over three days corresponding to baseline, intervention, and control periods were determined by analysis of 3DS using the Nutrition Data System for Research (NDSR) program developed by the University of Minnesota. NDSR is specifically designed for collecting 24-hour dietary recalls and analyzing records. The database is comprehensive with over 18,000 food including brand name products, menus items from restaurants, and ethnic foods. In addition, NDSR includes a dietary supplement assessment module. To assess compliance, the number of cereal bars eaten was tracked on a separate check list. Finally, periodic phone calls and emails throughout the study were used to ensure participant adherence to the dietary protocol and to address any questions or concerns.

### Anthropometrics

Body weight was measured using a “Health-O-Meter” scale and height was measured by stadiometer. Weight was measured initially, at baseline, and at the end of weeks 3, 6 and 9. Body mass index (BMI) (kg/m^2^) was determined and participants were classified as underweight (BMI < 18.5), normal weight (BMI 18.5–24.9), overweight (BMI 25.0–29.9) or obese (BMI>30) [10].

### Statistical Analysis

The primary study outcome measured was change of dietary calcium intake during the intervention. The secondary outcome measures were changes of energy intake and body weight during intervention. A priori power analysis was conducted using the G*Power (v. 3.1) test in order to determine an adequate sample size to detect statistical significance if significance in fact existed. In order to obtain a desired level of power at. 80, estimating a moderate effect of size (.25) and a one sided alpha level of. 05, a total of 28 participants was needed to test for between group differences of calcium and energy intake. Thus, the initial enrollment of 40 participants was considered sufficient. Data were analyzed using statistical software, Stata version 11.2. The Shapiro-Wilk test was used to test normality of the data. Data were not normally distributed and therefore, the Wilcoxon signed-rank test was used for within group comparisons of calcium and energy. Mann Whitney U test was used for baseline comparisons of demographic characteristics and for between group comparisons of calcium and energy. The Chi-squared test was used to compare ethnicity and supplement use. Differences were considered statistically significant if *P <0*.*05*.

## Results

### Participant Characteristics

Total of 40 individuals were consented to enroll in the study (Fig 1). Participants identified themselves as non-Hispanic White (34%), Asian (46%), non-Hispanic Black (14%), or Hispanic (6%) (Table 2). The average age was 27.8 years and ranged from 21 to 58 years. Weight classification based on BMI indicated that 14% of the participants were underweight, 69% normal weight, 14% overweight and 3% obese. Prior to randomization, 5 individuals withdrew (12.5%) for reasons unrelated to the study (4 scheduling conflicts, 1 relocation) (Fig 1). Participants then were randomly distributed into Group I (n = 18) or Group II (n = 17) (Fig 1). No significant differences were found by Mann Whitney U tests in age, height, weight, BMI, and age between the Groups I and II at randomization (Table 2).

**Table 2:** Baseline characteristics of All, Group I and Group II participants.^1^

	All	Group I	Group II	*P*
**Participants (n)** ^2^	35	18	17	
** Age (years)**	27 ± 7.9	28.2 ± 8.6	27.3 ± 7.4	0.58
** Height (cm)**	161.3 ± 5.7	160.1 ± 4.2	162.5 ± 6.9	0.18
** Weight (kg)**	57.5 ± 10.7	55.9 ± 9.8	59.1 ± 11.8	0.44
** BMI (kg/m2)**	22.0 ± 3.6	21.8 ± 3.1	22.4 ± 4.1	0.39
**Ethnicity (n) (%)** ^3^				
** Non-Hispanic White**	12 (34)	6 (33)	6 (35)	0.90
** Non-Hispanic Black**	5 (14)	2 (11)	3 (18)	0.58
** Hispanics**	2 (6)	2 (11)	0 (0)	0.16
** Asian**	16 (46)	8 (44)	8 (47)	0.88
**Supplementation** ^3^	6 (17)	3 (17)	3 (17)	0.94

^1^ Mean ±SD or number (percentage).

^2^ Mann Whitney U tests were used to test for baseline differences.

^3^ Chi-Square tests were used to compare ethnicity and supplementation use.

### Dietary Intake

Total mean daily calcium intake during the intervention period (1071 mg per day) was significantly higher compared to baseline (720 mg per day, *P <0*.*0001*) and control (775 mg per day, *P = 0*.*0001*) periods (Table 3). Similarly, Group I and Group II dietary calcium intake was significantly higher during intervention compared to baseline and control periods (*P*< 0.01) (Table 3). In contrast, total baseline and control calcium intake of all participants did not differ (Table 3). The percentage overall of all participants not meeting the RDA for calcium decreased from 88.6% from baseline to 60.0% during the cereal bar intervention. Cereal bar compliance was excellent (98.6%).

**Table 3:** Changes of anthropometrics, calcium and energy intake, and percentage not meeting calcium Recommended Dietary Allowances (RDA).^1^

	Group (n)	Baseline	Intervention	Control
**Weight (kg)**	All (35)	57.5 ± 10.7	58.1 ± 10.7	57.6 ± 10.6
	Group I (18)	55.7 ± 9.8	55.9 ± 7.1	56.2 ± 9.5
	Group II (17)	59.1 ± 11.6	59.1 ± 11.5	59.1 11.7
**BMI (kg/m2)**	All (35)	22.0 ± 3.6	22.3 ± 3.5	22.1 ± 3.5
	Group I (18)	21.7 ± 3.1	22.1 ± 3.2	21.9 ± 3.0
	Group II (17)	22.4 ± 4.1	22.6 ± 3.0	22.4 ± 4.1
**Energy (kcal/d)**	All (35)	1740 ± 531	1789 ± 492	1789 ± 579
	Group I (18)	1639 ± 445	1726 ± 441	1742 ± 552
	Group II (17)	1842 ± 616	1852 ± 543	1817 ± 606
**Calcium (mg/d)**	All (35)	732 ± 275	1092 ± 377 *	801 ± 373
	Group I (18)	714 ± 296	1044 ± 321 **†**	768 ± 339
	Group II (17)	725 ± 216	1097 ± 417 **‡**	783 ± 355
	Supplement (6)	806 ± 506	1153 ± 455 ¥	819 ± 516
**% not meeting RDA**	All (35)	88.6	60.0	74.2
	Group I (18)	88.9	61.1	72.2
	Group II (17)	70.6	23.5	58.8

^**1**^ Values are mean ±standard deviation or percentages.

* Difference from baseline and control (P < 0.0001).

**†** Different from baseline (P = 0.0003) and control (P = 0.02).

**‡** Different from baseline (P = 0.0003) and control (P = 0.0005).

¥ Different from baseline (P <0.0001) and control (P = 0.0001).

Most participants consumed the fortified bars as snacks during the day and reported eating smaller portions at meals. Only a few participants reported eating the bars as a replacement of breakfast items such as cereals and orange juice. NDSR output files were also created to compare major calcium contributions by food groups between the intervention and control periods for each participant. There was no significant difference in calcium contribution from milk/soy milk, cheese, yogurt, breakfast cereals, or entrees between the intervention and control periods. In addition, review of the 24-hour recalls for the two study periods did not reveal any consistent pattern of cereal bars substituting for specific foods or snacks.

Despite the consumption of 2 calcium-fortified cereal bars per day during the intervention period, no significant differences were found in the daily energy intake between baseline, intervention, and control periods (Table 3). However, participants daily average dietary fiber intake was significantly higher during the intervention (22.7 g/d) compared to control (19.3/d) (*P* = 0.006). One cereal bar contained 3 g of dietary fiber (Table 1).

### Body Weight and BMI

No overall significant changes in weight and BMI were found during the course of the study. However, 2 participants in Group II showed slight changes in weight categories. One participant experienced a slight weight increase from underweight (48.4 kg, BMI 18.3) to normal weight (49.3 kg, BMI 18.7). Another participant experienced weight fluctuations from an initial normal weight (52.9 kg, BMI 24.8) to overweight (53.2 kg, BMI 25,0) at the end of the baseline period, and then returned to normal weight (52.3 kg, BMI 24.5) by the end of the study.

### Supplementation

Participants who occasionally took supplements also increased total calcium intake during the intervention compared to the control period (P = 0.0001). The average daily calcium intake of supplement users was about 50 mg/d higher than non-supplement users (Table 3). Nevertheless, average calcium intake of supplement users was not significantly different from non-supplement users during baseline, intervention or control periods.

## Discussion

Dietary calcium intake was significantly greater when women consumed 2 fortified cereal bars daily for 3 weeks. Calcium intake of women was approximately 350 mg per day higher compared to usual dietary intakes. Furthermore, no difference was found in the calcium contribution from other major food sources between intervention and control periods. Therefore, we accepted the hypothesis that daily consumption of calcium-fortified whole grain cereal bars would increase calcium intake of healthy women. The potential utility of cereal bars to increase calcium intake is also supported by recent sale figures. In 2012, cereal bars and snack bars sales in the United States (US) totaled $3.7 billion. The growth of this food category has been largely due to the broad retail distribution of cereal bars, the increase of snack culture, and consumer interested in health and convenience in the US [11].

A second important study finding was that the calcium-fortified cereal bar intervention did not increase average energy intake. Moreover, weight changes over the 3 week intervention periods were also not significant. Therefore, we accepted the hypothesis that the addition of the calcium-fortified cereal bars to the usual diet of women would not significantly increase total daily calorie intake and result in weight gain. Although changes in satiety were not assessed in the study, consumption of 6 g/d of dietary fiber provided by 2 cereal bars may have increased satiety [12] and partially explain the lack of weight gain or increased calorie intake during the intervention. Replacement of energy dense snacks with cereal bars also could help to explain the lack of weight gain; however, no consistent pattern of cereal bars substituting for specific snack was found.

Previously, only a few intervention studies have used non-dairy calcium-fortified products to assess changes in calcium intake. Ready-to-eat breakfast cereal consumption without milk increased calcium intake 50 ±4 mg SEM/d [6]. Calcium fortified orange juices provides 350 mg of calcium per 240 mL serving and was used to increase calcium intake in a weight loss program [7]. In addition, soy milk is a popular substitute for dairy milk which is fortified with calcium (450 mg per 8 oz./240 mL) [8]. Soy milk was used as a non-dairy calcium intervention in postmenopausal Adventist women to assess bone health benefits.

Major strengths and limitations of this study should be noted. The primary strength was the use of a crossover design to examine the effectiveness of fortified cereal bars on increasing calcium intake. Fortified cereal bars provided a readily available vehicle to deliver additional calcium that was well accepted. Frequent contact by phone and email throughout the 9 week study also contributed to an excellent total compliance with the study protocol. In addition, calcium intake was estimated by nutrient analysis of 3DS records using a well-known research software program (Minnesota NDSR).

Limitations of the study include using 3DS to estimated calcium and energy intake which may not accurately reflect usual diet. Participants also were not a good representation of the general population in terms of ethnicity. Most study participants were either Asian (46%) or White (34%); however, Asians represent less than 5% of the US population in most places in the United States [13]. Furthermore, participants were mostly students and staff from the Texas Medical Center which also limits generalization. Finally, the length of the intervention (3 weeks) may not be sufficient time to sustain a long-term behavior change period of consuming calcium-fortified cereal bars. The study CONSORT checklist (S1 Checklist) is available as a supporting information file.

## Conclusion

Calcium intake of women was significantly increased when 2 fortified cereal bars were consumed daily for 3 weeks without increasing energy intake or body weight. Changes in calcium contribution from other good sources of dietary calcium were not found during the cereal bar intervention compared to the control period. Any future studies with calcium-fortified cereal bars should consider using a larger sample size, lengthen the duration of the intervention, determine calcium bioavailability, and possibly assess satiety, and monitor changes of bone health markers and bone density.

In summary, finding from this study demonstrated a feasible way to increase calcium intake of women while potentially providing an approach to help improve bone health and reduce the risk of osteoporosis. Additional research is warranted to determine if consumption of calcium fortified cereal bars helps to maintain and improve bone health.

## Supporting Information

S1 Checklist (PDF)

S1 Protocol (PDF)
