# Supplementary material for: Consumption of Calcium-Fortified Cereal Bars to Improve Dietary Calcium Intake of Healthy Women: Randomized Controlled Feasibility Study
Source: PLoS One. 2015 May 5;10(5):e0125207. doi: 10.1371/journal.pone.0125207 (PMC4420267; doi:10.1371/journal.pone.0125207)
Supplement: S1 Protocol — (PDF) [file pone.0125207.s002.pdf]

TEXAS WOMAN'S UNIVERSITY
DENTON DALLAS HOUSTON

Office of Research
6700 Fannin Street
Houston, TX 77030-2343
713-794-2480 Fax 713-794-2488

May 5, 2014

Ms. Jennifer Lee
Nutrition and Food Sciences
6700 Fannin Street
Houston, TX 77030

Dear Ms. Lee:

Re: Effect of Calcium-fortified Cereal Bars on Dietary Calcium Intake in Women (Protocol #: 16802)

The TWU Institutional Review Board (IRB) has received the materials necessary to complete the file for the above referenced study approved on 9/30/2013. As applicable, agency approval letter(s), the final report, and signatures of the participants have been placed on file. As of this date, this protocol file has been closed.

Sincerely,

Jan Foster, PhD, APRN, CNS
Institutional Review Board - Houston

cc. Ms. Rose Bush, Department of Nutrition & Food Sciences - Houston
John Radcliffe, PhD, RD, Department of Nutrition & Food Sciences - Houston

TWU
EXAS WOMAN'S UNIVERSITY
DENTON DALLAS HOUSTON

Office of Research
6700 Fannin Street
Houston, TX 77030-2343
713-794-2480 Fax 713-794-2488

November 16, 2011

Ms. Jennifer Lee
Nutrition and Food Sciences
6700 Fannin Street
Houston, TX 77030

Dear Ms. Lee:

Re: *"Effect of Calcium-fortified Cereal Bars on Dietary Calcium Intake in Women" (Protocol #: 16802)*

Your application to the IRB has been reviewed and approved.

This approval lasts for one (1) year. The study may not continue after the approval period without additional IRB review and approval for continuation. It is your responsibility to assure that this study is not conducted beyond the expiration date.

Any modifications to this study must be submitted for review to the IRB using the Modification Request Form. Additionally, the IRB must be notified immediately of any unanticipated incidents. If you have any questions, please contact the TWU IRB.

The signed consent forms, as applicable, and final report must be filed with the Institutional Review Board in the Office of Research, IHS 10110, at the completion of the study.

Sincerely,

Carolyn Kelley, PT

Carolyn Kelley, PT, DSc, NCS
Institutional Review Board - Houston

TWU
TEXAS WOMAN'S UNIVERSITY
DENTON DALLAS HOUSTON

Office of Research
6700 Fannin Street
Houston, TX 77030-2343
713-794-2480 Fax 713-794-2488

October 18, 2012

Ms. Jennifer Lee
Nutrition and Food Sciences
6700 Fannin Street
Houston, TX 77030

Dear Ms. Lee:

Re: Effect of Calcium-fortified Cereal Bars on Dietary Calcium Intake in Women (Protocol #: 16802)

The request for an extension of your IRB approval for the above referenced study has been reviewed by the TWU Institutional Review Board (IRB) and appears to meet our requirements for the protection of individuals' rights.

If applicable, agency approval letters must be submitted to the IRB upon receipt PRIOR to any data collection at that agency. A copy of all signed consent forms and an annual/final report must be filed with the Institutional Review Board at the completion of the study. A copy of the approved consent form with the IRB approval stamp is enclosed. Please use a copy of this stamped consent form when obtaining consent from your participants.

This extension is valid one year from October 18, 2012. Any modifications to this study must be submitted for review to the IRB using the Modification Request Form. Additionally, the IRB must be notified immediately of any unanticipated incidents. If you have any questions, please contact the TWU IRB.

Sincerely,

Carolyn Kelley, PT

Carolyn Kelley, PT, DSc, NCS
Institutional Review Board - Houston

Texas Woman's University Institutional Review Board

Application for Expedited and Full Review

Name of Principal Investigator (PI): Jennifer Lee Phone: 281-782-8789

Status: ☐ faculty ☒ student ☐ staff ☐ other: ____ E-mail: jlee9@twu.edu

Address where correspondence is to be sent: 2111 Holly Hall St. APT811
Houston, TX 77054

Title of Study: Effect of Calcium-fortified Cereal Bars on Dietary Calcium Intake in Women

If the PI is a student, provide the following information:

Colleague ID #: 0970519 Department: Nutrition and Food Sciences

Name & Phone # of Research Advisor: John Radcliffe, PhD, RD, 713-794-2375

Estimated beginning date of study: 11/28/2011 Estimated duration of study 1 year

Campus (Denton, Dallas, or Houston) Houston Level of review: ☒ expedited ☐ full

Type of Project: (check all that apply)
☒ thesis ☐ professional paper ☐ dissertation
☐ class project ☐ faculty research ☐ pilot
☐ funded project (funding source: ____)
☐ other ____

Signatures:

Principal Investigator (PI): Signature certifies that the investigator has primary responsibility for all aspects of the research project.

Jenny Lee 11/11/2011
Principal Investigator Date

Faculty Research Advisor (for student research only): Signature certifies that the faculty member has read, reviewed, and approved the content of the application and is responsible for the supervision of this research study.

John Radcliffe 11 November 2011
Faculty Research Advisor Date

Academic Administrator: Signature certifies that the administrator has read, reviewed, and approved the content of the application.

[illegible] 11/11/2011
Academic Administrator (Department Chair, Program Director, or Associate Dean) Date

METHODOLOGY

Please refer to instructions when completing this form.

1. **Describe the purpose of study, including research questions and/or hypotheses.**
The purpose of this study is to determine the effect of consuming calcium-fortified cereal bars on calcium intake in women. The average intake of calcium in women in the United States from the diet (about 800 mg/day) is below the Recommended Dietary Allowance (1000 mg/day for ages 18 to 50 years, 1200 mg/day for age above 50 years), which increases their risk of developing osteoporosis. Consumption of two calcium-fortified cereal bars per day may be a feasible way to increase the dietary intake of calcium. This study will use Kellogg's Nutri-Grain® cereal bars, which provide 200 mg of calcium per bar, or a total of 400 mg/day.

2. **Participant Information:**

a. Description of participants in study:
The participants will be healthy adult women over the age of 18, able to speak, read, and understand English, and able and willing to consume Kellogg's Nutri-Grain® cereal bars and keep diet and supplement diaries. They will not be pregnant or planning to become pregnant during the course of the study, and not consuming calcium-containing medications or calcium supplements. They will not be regularly consuming more than two Kellogg's Nutri-Grain® cereal bars per month.

b. Approximate number of participants: 40

c. Vulnerable populations as participants (check all that apply):

Prisoners .... ☐
Pregnant women.................................. ☐
Fetuses / neonates .... ☐
Minors.. ☐

NOTE: Researchers must comply with the federal mandate to report child abuse. See instructions for details.

d. Age (or age range) of participants: Ages 18 years and older

Provide the rationale for inclusion/exclusion on the basis of age:
The focus of this study is on the effect of consuming cereal bars fortified with calcium in adult women.

e. Sex of participants .... ☐ Male ☒ Female ☐ Both

Provide the rationale for inclusion/exclusion on the basis of sex:
The participants of this study will be women. In general, women are more susceptible to osteoporosis than men. According to the National Osteoporosis Foundation, about 80% of the 44 million Americans affected by osteoporosis and low bone mass are women.

f. Participants will be excluded based on ethnicity: .... ☐ Yes ☒ No

If yes, provide a description of the exclusion criteria and the rationale for using these criteria:

g. List and provide rationale for any other inclusion/exclusion criteria:
Inclusion criteria: Able to read, write, speak, and understand English so as to be able to communicate with the Principal Investigator, and be able to keep diet and supplement diaries, in which they are able to record all foods, drinks, and supplements consumed.

Exclusion criteria: Unable or unwilling to consume Kellogg's Nutri-Grain® cereal bars or being allergic to any of the ingredients of this product, as this would mean that the food product would not be consumed. The ingredients are: oats, wheat flour, wheat bran, wheat gluten, corn fiber, cornstarch, corn syrup, whey, soybean oil, soy lecithin, sugar, red #40, strawberries, blueberries, apples, and cinnamon; taking any supplements or medications containing calcium; regularly consuming more than two Kellogg's Nutri-Grain® cereal bars per month; being pregnant or planning to become pregnant during the course of the study, as pregnancy may be associated with changes in food preferences and intake, and may cause nausea and vomiting that may limit the ability to consume the bars given to the participants; taking medications, as this may alter taste and/or food intake; having liver disease, kidney disease, gastrointestinal disease, a history of bariatric surgery, having had a cardiovascular event (stroke or myocardial infarction), as these conditions may affect food intake; undergoing treatment for non-melanoma skin cancer, as this may affect food intake; being on a weight control diet, a disease specific diet, as this may limit food intake; being on a vegan diet, as the cereal bars contain ingredients of animal origin; having a diagnosed eating disorder, as this may affect food intake; taking calcium supplements or calcium-containing medications, as the purpose of this study is to determine the impact of consuming cereal bars fortified with calcium on the intake of calcium.

3. Describe the participant recruitment process in detail. Attach any recruitment materials or scripts.

The participants will be recruited by posting flyers at the Texas Woman's University (TWU), Houston Center. The flyer (See Appendix 1) will ask potential participants to contact the Principal Investigator. Permission to post the flyers has been obtained (see Appendix 2). Potential participants interested in participating in the study will be asked to visit the Principal Investigator at TWU, Houston Center. A description of what is involved with the study will be given (see Appendix 3), and an informed consent will be completed.

4. Describe in detail the research procedures.

Potential participants will have this nine-week study explained to them by the Principal Investigator, who will also obtain consent from the participants, collect data on age, height, weight, and ethnicity at the beginning of the study, and measure their weights again at weeks 3, 6, and 9 of the study (see Appendix 4). These activities will take place in Room 10132B at TWU, Houston Center, with the door closed, and only the Principal Investigator and the participant will be present. Height and weight will be measured using an instrument called a Health-O-Meter. Participants will be fully clothed only with shoes removed. The participants will be randomly assigned into either Group I or II by drawing of numbers from a hat. Numbers 1 to 20 will be in Group I, and numbers 21 to 40 will be in Group II. The participants in Group I will be asked to consume their usual diet for 3 weeks. After this, each participant will be given 42 Kellogg's Nutri-Grain® cereal bars to be consumed for the following 3 weeks, and then they will be asked to consume their usual diet for another 3 weeks. The participants in Group II will be asked to consume their usual diet for 6 weeks, and then each person be given 42 Kellogg's Nutri-Grain® cereal bars to be consumed for the following 3 weeks. All participants will be asked to complete a three-day diet and supplement diary (see Appendix 5) for weeks 2, 5, and 8. Instructions on how to keep a diet and supplement diary will be given. During the three-week period when participants are asked to consume the cereal bars, the participants will be asked to keep a checklist

to record the numbered bars consumed each day (see Appendix 6). Participants will be asked to meet with the Principal Investigator once every 3 weeks. A schedule with the date of the subsequent meetings will be given (see Appendix 7). Completed diet and supplement diaries will be returned to the Principal Investigator and be reviewed during subsequent meetings. Only the participant and the Principal Investigator will be present when the instructions on how to keep a diet and supplement diary are given, the diet and supplement diaries are distributed, and the completed diet and supplement diaries are reviewed. Participants will be asked to return any uneaten bars. All these activities will take place in Room 10132B at TWU, Houston Center.

Coded diet and supplement diaries will be analyzed for the intake of calcium using the Minnesota Data Analysis Software and the statistical analysis of the coded data will be carried out by the Principal Investigator in a computer in Room 10128 at TWU, Houston Center. A hard copy of the results of the statistical analysis will be obtained, and no coded data will be stored in this computer.

a. Is video recording a part of the study? .... ☐Yes ☒ No

With sound ☐ Without sound ☐

b. Is audio recording a part of the study? .... ☐Yes ☒ No

If you answered "yes" to question #4a or 4b, describe the purpose of the recording and who will have access to these recordings.

c. Is internet / email a part of the study? .... ☐Yes ☒ No

If you answered "yes" to question #4c, describe how the internet and/or email will be used.

5. What is the time commitment for the participants? Include the number of sessions, maximum time commitment per session, and the maximum cumulative time commitment.

At the first meeting, instructions will be given on how to keep a diet and supplement diary. This should take no more than 15 minutes. The participants will be asked to keep one three-day diet and supplement diary on the second week of each of the three three-week periods of the study. Keeping the diet and supplement diaries should take no more than 10 minutes per day, 30 minutes per week, or 1 hour and 30 minutes for the entire study. The participants will be asked to record the consumption of the numbered cereal bars using a check list for each of the 21 days of the study when they are asked to consume the bars. This should take no more than 1 minute per day, 7 minutes per week, or 21 minutes for the whole study. The diet and supplement diaries will be reviewed during meetings with the Principal Investigator at the end of weeks 3, 6, and 9 of the study. Each review meeting should take no more than 15 minutes, giving a total of 45 minutes for the entire study. Weight and height of each participant will be taken at the beginning of the study. This should take no more than 3 minutes. Their weight will be taken again at the end of weeks 3, 6, and 9. This should take no more than 2 minutes per session for a total of 6 minutes for the study. For receiving instructions, keeping the diet and supplement diaries, recording consumption of numbered cereal bars, having the diet and supplement diaries reviewed, and having height and weight measured, the total time commitment of each participant will be no more than 3 hours.

6. Site / location of the study.

a. Will participants be affiliated with a specific non-TWU agency, institution, or organization? .... ☐ **Yes** ☒ **No**

If yes:

Name of the site(s)?

*Affiliation of the **principal investigator** to this site(s)?*

*Affiliation of the **participants** to this site(s)?*

Agency approval letters are required by the IRB before data can be collected at a site. If you answered "yes" to 6a, attach the signed agency approval letter on letterhead from each agency. If agency approval cannot be obtained prior to submitting the IRB application, explain here.

b. Describe the setting of the study (i.e. physical location, surroundings, privacy aspects, etc.)
The study will take place at TWU, Houston Center. The explanation of the study, the consenting of the participants, the collection of the data on age, height, and weight, the distribution of the cereal bars, and the distribution and reviewing of diet and supplement diaries will take place in Room 10132B by the Principal Investigator. Analysis of the diet and supplement diaries and the statistical analysis will be carried out in Room 10128 by the Principal Investigator.

POTENTIAL RISKS AND PROTECTION OF PARTICIPANTS

7. Explain the potential risks to the human participants involved in this research. All risks must be identified and listed on the consent form (if applicable).

RISK	STEPS TO MINIMIZE RISK
Loss of confidentiality	Confidentiality will be maintained by using codes rather than names. The data will be stored in a locked filing cabinet in Room 10132B at the TWU, Houston Center. Only the Principal Investigator and the advisor for this study will have access to the filing cabinet. All data will be destroyed by shredding before 09/01/2021. All meeting sessions will be held in a private setting in Room 10132B or a location agreed upon by the Principal Investigator and the participant.
RISK	STEPS TO MINIMIZE RISK
Weight gain	The potential for weight gain can be avoided by letting participants know that the bars to be consumed will provide approximately 240 kilocalories per day. Participants may elect to eat less of the other foods that they normally eat to maintain usual intake and body weight.
RISK	STEPS TO MINIMIZE RISK

Disruption of schedule	Visits will be scheduled at times convenient for the participants at TWU, Houston Center, to accommodate individual schedules.

(Use continuation pages if necessary)

8. Will participants be told about the intent of the study prior to participating? ...☒ Yes ☐ No
If "no," provide an explanation of why deception is necessary and the debriefing method to be used to fully inform the participants of the study's intent.

9. Explain when and how the participants will be given the opportunity to ask questions.
The participants will be given the opportunity to ask questions about the study during the time the script is being read to them by the Principal Investigator, and when the Principal Investigator has finished reading the script. They may also ask questions when they are reading the informed consent and any time after signing the informed consent until the end of the study. The phone numbers and e-mail addresses of the Principal Investigator and the advisor of the study will be given to the participants in the informed consent. The participants will be asked if they have any questions before and after each meeting session, and they will be encouraged to contact the Principal Investigator at any time with any questions or concerns regarding the study.

10. Identifiable Data

Outline the steps to ensure the confidentiality of <u>identifiable</u> data. Identifiable data includes documents, audio and video recordings, electronic data, and blood or other human specimens.

a. Explain what identifiable data, if any, will be collected.
Data on the intake of food, drinks, and supplements, age, height, weight and ethnicity will be collected in this study.

b. Where will identifiable data be stored? (Specify precise location, preferably in a locked file cabinet with limited access.)
All written documents will be stored in a locked cabinet in Room 10132B in TWU, Houston Center.

c. Give the date that identifiable data will be destroyed (mm/dd/yy). If identifiable data will be stored for an indefinite period of time, please explain.
All identifiable data will be destroyed on 09/01/2021.

d. Identify specific ways that identifiable data will be destroyed at the end of this period of time.
All identifiable data will be destroyed by shredding.

e. Because the academic component of TWU is classified as a non-covered HIPAA entity, identifiable health or health-related data cannot be transmitted electronically. You must be able to answer "no" to at least one of the following questions in order for your study to be approved.

Does this research involve health or health-related data? .... ☒ Yes ☐ No
If yes, are the data identifiable? .... ☒ Yes ☐ No
If yes, will data be transmitted electronically? .... ☐ Yes ☒ No

BENEFITS/REMUNERATION

11. **What will the participant receive for taking part in the study (*i.e., financial remuneration, free services, access to information, and access to an intervention)*?**
The participants will directly benefit from their participation by receiving $20 gift certificate upon completion of the study. Participants will receive free food as cereal bars from the study.

12. **What are the generalizable benefits of this study? *(e.g., contribution to knowledge in field).***
The study may demonstrate the feasibility of using calcium-fortified cereal bars to increase calcium intake for the prevention of osteoporosis.

13. **Explain when and how the participants will be provided with the results of the study.**
After data have been analyzed and evaluated, a summary of the major findings will be mailed to participants who request that this information be mailed to them by providing the researchers with their address in the informed consent.

INFORMED CONSENT PROCEDURES

14. **If you will use written informed consent, explain how that consent will be obtained and attach a copy of the consent form.**
Potential participants will be given a copy of the informed consent. They will have the opportunity to ask questions about the consent form. If they understand the material in the consent form and they wish to participate in the study, they will be asked to initial pages 1-2 and to sign page 3 of the consent form. A copy of the informed consent is attached (see Appendix 8).

15. **If you will not use written informed consent, provide a detailed rationale and explain how informed consent will be obtained.**

RESEARCH TEAM MEMBERS

16. **Provide a list of all research team members and their role on the project. Attach copies of current training certificates.**

Name of Team Member	Role on Project	Training Certificate Attached
Jennifer Lee	Principal Investigator	☒
John Radcliffe	Advisor	☒
		☐
		☐
		☐
		☐
		☐
		☐

ATTACHMENTS

17. **Provide a list of all attachments to this application in the order cited on this form.**

Appendix 1: Flyer
Appendix 2: Permission to post flyer
Appendix 3: Script to potential participants
Appendix 4: Age, ethnicity, height, and weight

PARTICIPANTS NEEDED!!

Texas Woman's University - Houston Center

9 WEEKS FEEDING STUDY

The purpose of this study is to determine if eating calcium-fortified cereal bars will result in higher intakes of calcium.

IF YOU ARE:

- Healthy woman
- Age 18 years or older
- Not taking calcium-containing medications or calcium supplements
- Not allergic to dairy, soy, wheat
- Not pregnant nor planning to become pregnant during the course of the study
- Not eating more than 2 Kellogg's Nutri-Grain® cereal bars per month

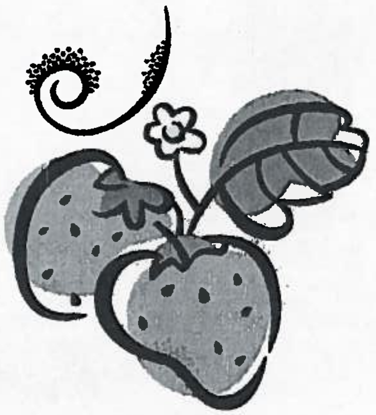

YOU WILL BE ASKED TO:

- Make 4 visits to the Texas Woman's University, Houston Center
- Either consume your usual diet for 6 weeks then receive cereal bars for the next 3 weeks or consume your usual diet for 3 weeks, receive cereal bars for 3 weeks, then consume your usual diet again for 3 weeks
- Have your height taken on one occasion and weight taken on four occasions
- Eat 2 Kellogg's Nutri-Grain® cereal bars per day for 3 weeks for FREE!

Participants will receive $20 gift card upon completion of this study.

Parking will be reimbursed.

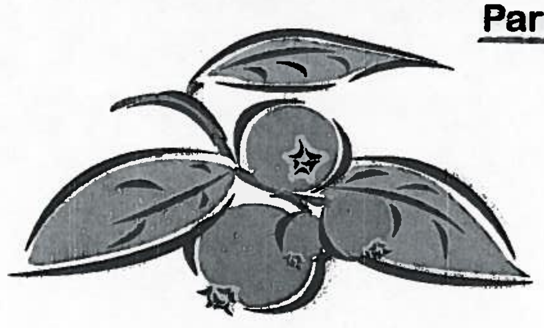

For More Information, Please Contact:

Jennifer Lee

281-782-8789

Jennifer Lee
281 782 8789
JLEE9@TWU.EDU

Jennifer Lee
281 782 8789
JLEE9@TWU.EDU

Jennifer Lee
281 782 8789
JLEE9@TWU.EDU

Jennifer Lee
281 782 8789
JLEE9@TWU.EDU

Jennifer Lee
281 782 8789
JLEE9@TWU.EDU

Jennifer Lee
281 782 8789
JLEE9@TWU.EDU

Jennifer Lee
281 782 8789
JLEE9@TWU.EDU

Jennifer Lee
281 782 8789
JLEE9@TWU.EDU

Jennifer Lee
281 782 8789
JLEE9@TWU.EDU

Jennifer Lee
281 782 8789
JLEE9@TWU.EDU

Jennifer Lee
281 782 8789
JLEE9@TWU.EDU

Jennifer Lee
281 782 8789
JLEE9@TWU.EDU

APPENDIX 2

PERMISSION TO POST FLYER

TWU
TEXAS WOMAN'S UNIVERSITY
DENTON DALLAS HOUSTON

Institute of Health Sciences-Houston Center
Office of Student Life, Suite 2300
6700 Fannin St., Houston, TX 77030-2343
713-794-2157 FAX 713-794-2169 www.twu.edu/student-life-houston/

November 11th, 2011

To Whom It May Concern:

For a study entitled, "The Effect of Calcium-Fortified Cereal Bars on Dietary Calcium Intake in Women", Jennifer Lee, a Nutrition Master's student, is approved to place a flyer advertising the study on the third floor bulletin board in the vending machine area during the period of November 28, 2011 through April 28th, 2012.

Respectfully,

Deborah Unruh

Deborah Unruh, Coordinator

Office of Student Life

APPENDIX 3

SCRIPT FOR POTENTIAL PARTICIPANTS

SCRIPT FOR POTENTIAL PARTICIPANTS

Hello, I am Jennifer Lee, a graduate student at Texas Woman's University (TWU), Houston Center.

Thank you for your interest in this study. If you have any questions after I begin explaining the study, please let me know. In order to take part in this study, you must be female and aged 18 years or older. You must be able to speak, read, and understand English. You must be able to keep a three-day diet and supplement dairy. You must be able to consume Kellogg's Nutri-Grain® cereal bars. You should not be taking any calcium-containing supplements or medications. You should not be consuming more than two Kellogg's Nutri-Grain® cereal bars per month. You must not be pregnant or plan to become pregnant during this nine-week study. You must not be allergic to any ingredients in this food.

The purpose of this nine-week study is to determine if the consumption of Kellogg's Nutri-Grain® cereal bars fortified with calcium will increase calcium intake in women. Calcium intake in the United States is below recommended levels and eating cereal bars such as Kellogg's Nutri-Grain® cereal bars may be a way to increase calcium intake.

Participants in the study will be assigned to either Group I or II. If you are assigned to Group I, you will be asked to consume your usual diet for three weeks, be given 42 Kellogg's Nutri-Grain® cereal bars to be consumed for the following three weeks, and be asked to consume your usual diet again for another three weeks. If you are assigned to Group II, you will be asked to consume your usual diet for six weeks, and then be given 42 Kellog's Nutri-Grain® cereal bars to be consumed for the following three weeks. You will be asked to consume 2 bars per day for these 3 weeks.

Regardless of the group that you are in, you will have your height and weight taken at the beginning of the study, and your weight taken again at weeks 3, 6, and 9 of the study. You will need to make four visits to TWU. Your total time commitment for the study will be no more than 3 hours. This includes time to have our height and weight taken, to receive instructions on how to keep a diet and supplement diary, and to record your intake of foods and drinks for 9 days of the study.

If you need to park in one of the parking garages in the Texas Medical Center in order to participate in this research study, you will be reimbursed in cash the day of your visit. Upon completion of the study, you will receive a gift certificate for $20.

In order to preserve confidentiality for this research study, you will be given a code number. All records will be kept in a locked filing cabinet in Room 10132B in Texas Woman's University, Houston Center. Only Jennifer Lee (a graduate student at TWU) and John Radcliffe, PhD, RD will have access to the cabinet.

Now I would like to ask you if you have any questions about the study. If you do not have any questions, or if I can answer your questions, and you are still interested in the study, I will give you a copy of a form called an informed consent to read. You should read this form carefully. If you have any questions about the informed consent, you should ask me to make the meaning clearer. If you do not have any questions about the form, or I answered all your questions about the form, you will be asked to sign the consent form.

Do you have any questions?

I will review the consent form with you, as I do so, please ask me if you have any questions about it.

Thank you so much for your interest in the study and taking the time to come and talk to me about it.

APPENDIX 4

AGE, ETHNICITY, HEIGHT, WEIGHT

Code Number: ____________________________

Age in years: ________

Height: _______________ Date:________

Weight: _______________Date:________

Ethnicity:

- ☐ Non-Hispanic White
- ☐ Non-Hispanic Black
- ☐ Mexican-American/Hispanic
- ☐ Asian
- ☐ Others __________________________

Weight: _______________Date:________

Weight: _______________Date:________

Weight: _______________Date:________

APPENDIX 5

DIET AND SUPPLEMENT DIARY FORM

Code Number: ____________________ Date: ____________________

Time	Brand Name	Food Item / Supplement	Type	Amount
			□ Regular<br>□ Low-Fat ________%<br>□ Fat Free<br>□ Other: __________	
			□ Regular<br>□ Low-Fat ________%<br>□ Fat Free<br>□ Other: __________	
			□ Regular<br>□ Low-Fat ________%<br>□ Fat Free<br>□ Other: __________	
			□ Regular<br>□ Low-Fat ________%<br>□ Fat Free<br>□ Other: __________	
			□ Regular<br>□ Low-Fat ________%<br>□ Fat Free<br>□ Other: __________	
			□ Regular<br>□ Low-Fat ________%<br>□ Fat Free<br>□ Other: __________	
			□ Regular<br>□ Low-Fat ________%<br>□ Fat Free<br>□ Other: __________	
			□ Regular<br>□ Low-Fat ________%<br>□ Fat Free<br>□ Other: __________	
			□ Regular<br>□ Low-Fat ________%<br>□ Fat Free<br>□ Other: __________	
			□ Regular<br>□ Low-Fat ________%<br>□ Fat Free<br>□ Other: __________	

APPENDIX 6

CHECKLIST FOR CONSUMPTION OF NUMBERED BARS

Code Number: ______________________

CHECKLIST

Week _____

Date	Bar Number	
	☐ 1	☐ 2
	☐ 3	☐ 4
	☐ 5	☐ 6
	☐ 7	☐ 8
	☐ 9	☐ 10
	☐ 11	☐ 12
	☐ 13	☐ 14

Week _____

Date	Bar Number	
	☐ 15	☐ 16
	☐ 17	☐ 18
	☐ 19	☐ 20
	☐ 21	☐ 22
	☐ 23	☐ 24
	☐ 25	☐ 26
	☐ 27	☐ 28

Week _____

Date	Bar Number	
	☐ 29	☐ 30
	☐ 31	☐ 32
	☐ 33	☐ 34
	☐ 35	☐ 36
	☐ 37	☐ 38
	☐ 39	☐ 40
	☐ 41	☐ 42

Code Number: ____________________ Date: ____________________

GROUP I SCHEDULE

Purpose of Visit:

Visits	Date	Instruction/ Consent to Participate	Group Assignment	Height Taken	Weight Taken	Pick up Cereal Bars	Pick up Blank Diaries	Return & Review Previous Diaries	Additional Comments
1		√	√	√	√		√		
2					√	√	√	√	
3					√		√	√	
4					√			√	

Code Number: ______________________   Date: ____________________

GROUP II SCHEDULE

Purpose of Visit:

Visits	Date	Instruction/ Consent to Participate	Group Assignment	Height Taken	Weight Taken	Pick up Cereal Bars	Pick up Blank Diaries	Return & Review Previous Diaries	Additional Comments
1		√	√	√	√		√		
2					√		√	√	
3					√	√	√	√	
4					√			√	

APPENDIX 8

CONSENT FORM

TEXAS WOMAN'S UNIVERSITY
CONSENT TO PARTICIPATE IN RESEARCH

Title: Effect of Calcium-fortified Cereal Bars on Dietary Calcium Intake in Women

Investigator: Jennifer Lee ....jlee9@twu.edu 281/782-8789
Advisor: John Radcliffe, PhD, RD...jradcliffe@twu.edu 713/794-2375

Explanation and Purpose of the Research

You are being asked to participate in the research study for Ms. Lee's thesis at Texas Woman's University (TWU), Houston Center. The intake of calcium from the diet for adult women in the United States is below the recommended level, which increases their risk of developing osteoporosis. The purpose of this study is to determine whether eating Kellogg's Nutri-Grain® cereal bars, providing approximately 40% of the daily need for calcium, will increase calcium intake in healthy adult women.

Your calcium intake will be calculated from information from your diet diaries that you will be asked to keep. A nutrient analysis program will be used. Your intake of energy (as calories), fat, protein, carbohydrates, vitamins, and minerals will also be determined to help describe the effect of consuming cereal bars on your nutritional status.

In order to take part in this study, you must be over the age of 18 years, be able to speak, read, and understand English, be able to keep three-day diet diaries, and be able to eat Kellogg's Nutri-Grain® cereal bars and not be allergic to any of the ingredients (oats, wheat flour, wheat bran, wheat gluten, corn fiber, cornstarch, corn syrup, whey, soybean oil, soy lecithin, sugar, red #40, strawberries, blueberries, apples, and cinnamon). You should not be regularly consuming more than two Kellogg's Nutri-Grain® cereal bars per month. You must not be pregnant or plan to become pregnant during this nine-week study. You must not be taking any calcium-containing medication. You must not have kidney disease, liver disease, gastrointestinal disease (celiac disease, ulcerative colitis, and Crohn's disease), a history of bariatric surgery, have had a major cardiovascular event (stroke or myocardial infarction), be undergoing treatment for cancer with the exception of non-melanoma skin cancer, be following a weight control diet or a disease specific diet, be following a vegan diet, or have a diagnosed eating disorder.

Description of Procedures

You will be one of 40 participants in this nine-week study. As participants, you will be placed into either Group I or Group II by drawing numbers from a hat. If you are assigned to Group I, you will be asked to eat your usual diet for the first three weeks of the study, then you will be given 42 cereal bars to be consumed for the following three weeks, with two bars being available each day; after this, you will be asked to eat your usual diet again for the next three weeks. If you are assigned to Group II, you will be asked to eat your usual diet for the first six weeks of the study, and then you will be given 42 cereal bars to be consumed for the following three weeks. You will be asked to consume 2 bars per day for three weeks. You will be asked

to keep a three-day diet and supplement diary on weeks 2, 5, and 8 of the study. You will be asked to keep a daily check list of cereal bars consumed during the three weeks when the cereal bars are made available to you. You will be asked to have four visits to the Department of Nutrition and Food Sciences at TWU, Houston Center. Cereal bars will be given to you as necessary during these visits. You will be asked to return any uneaten cereal bars.

During your first visit, after completing the consent form, you will be assigned to either Group I or Group II. You will be assigned a code number. You will have your height and weight taken, and you will be asked to give your age and ethnicity. You will have your height and weight taken using an instrument called a Health-O-Meter. You will be given a schedule with dates and times for your next meeting session, blank pages of the diet diaries with your code number, and instructions how to keep a three-day diet and supplement diary. You will need to keep a three-day diet and supplement diary during weeks 2, 5, and 8 of the study, each with two nonconsecutive (not next to each other) weekdays and one weekend day. However, you may choose to have a weekday next to a weekend day (for example, a Friday next to a Saturday or a Sunday next to a Monday). You will be asked to write down the brand name, type of food, amount consumed, and the time it was consumed (for example, HEB, Milk, 2% fat, 1 glass [8 oz], 8 am), as well as the names of any supplements (for example, Centrum). You will be provided with measuring cups that will enable you to estimate the amounts of foods and drinks that you consume. However, you may record commonly used items without using the measuring cups (for example, one can of Sprite). Also, you may only need to measure the amount(s) of the food item(s) consumed once if you eat similar amount(s) each day. You will be asked to complete a check list during each of the weeks when you are given the cereal bars.

During the second, third, and fourth meeting sessions (after the third, sixth, and ninth week of the study), you will return the completed three-day diet and supplement diaries to the researcher and you will have your diet and supplement diary reviewed with the researcher. You will be given blank diet and supplement diaries during the meeting sessions at the third and sixth weeks of the study. If you are in Group I, you will be given 42 cereal bars during the second meeting (after the third week of the study). If you are in Group II, you will be given 42 cereal bars during the third meeting session (after the sixth week of the study). You will be asked to have your weight measured again at the end of weeks 3, 6, and 9.

It is estimated that your total time commitment for the study will be no more than 3 hours. It will take no more than 3 minutes to have your height and weight taken on your first visit and no more than 6 minutes to have your weight taken at three subsequent visits (3x2 minutes); it will take no more than 15 minutes to receive instructions on how to keep a diet and supplement diary; it should take no more than 10 minutes per day, 30 minutes per week, or a total of 1 hour and 30 minutes for weeks 2, 5, 8 of the study to keep your diet and supplement diaries; it should take you no more than 1 minute per day, 7 minutes per week, or 21 minutes per 3 weeks to keep a check list during the period when you are given cereal bars; it should take no more than 15 minutes per session, and 45 minutes per 3 sessions to have your diet diaries reviewed at the end of weeks 3, 6, and 9.

You will receive cash reimbursement if you park at TWU at the day of your visit.

______
Participant Initials

Potential Risks

You will be asked to consume two Kellogg's Nutri-Grain® cereal bars per day, providing approximately 240 kilocalories per day, so a potential risk for this study is weight gain. However, the potential for weight gain may be reduced, as you will most likely eat less of other food items that you normally eat, and your weight should not change.

Another risk in this study is loss of confidentiality. However, every effort will be made to maintain the confidentiality of the study records. Confidentiality will be maintained by using codes rather than names. The data will be stored in a locked filing cabinet in Room 10132B. Only the researcher and the advisor of this study will have access to the filing cabinet. All data will be destroyed by shredding before September 1st, 2021. All meeting sessions will be held in Room 10132B at TWU, Houston Center or at a private setting agreed upon by you and the researcher. The data and results of the study may be reported in scientific magazines or journals but your name or any other identifying information will not be included. Confidentiality will be protected to the extent allowed by law.

There is a risk that your normal schedule may be disrupted by coming to TWU, Houston Center. To minimize this risk, the meeting sessions can be scheduled within an 8-hour period (from 9 am to 5 pm) during week days.

The researchers will try to prevent any problem that could happen because of this study. You should let the researchers know at once if there is a problem and they will help you. However, TWU does not provide medical services or financial assistance for injuries that might happen because you are taking part in this research.

Participation and Benefits

Your involvement in this study is completely voluntary and you may withdraw from the study at any time. Following the completion of the study, you will receive a $20 gift card for your participation. Another benefit is that you will receive free food in the form of 42 Kellogg's Nutri-Grain® cereal bars valued at $1 per bar. This study may also provide you with knowledge of the potential health benefits of consuming cereal bars fortified with calcium. If you would like to know the results of this study, we will mail them to you.*

Questions Regarding the Study

You will be given a copy of this signed and dated consent form to keep. If you have any questions about the research study you could ask the researchers at any time during the entire course of the study; their phone numbers are at the top of this form. If you have questions about your rights as a participant in this research or the way this study has been conducted, you may contact the Texas Woman's University Office of Research at 713-794-2480 or via e-mail at IRB@twu.edu.

____ ________________

Signature of Participant Date

*If you would like to know the results of this study tell us the address of where you want them to be sent:

E-mail: ______________________________

Or address: ______________________________

____

Certificate of Completion

The National Institutes of Health (NIH) Office of Extramural Research certifies that **Jennifer Lee** successfully completed the NIH Web-based training course "Protecting Human Research Participants".

Date of completion: 09/07/2010

Certification Number: 505624

Certificate of Completion

The National Institutes of Health (NIH) Office of Extramural Research certifies that **John Radcliffe** successfully completed the NIH Web-based training course "Protecting Human Research Participants".

Date of completion: 07/31/2011

Certification Number: 506753
